# Intraperitoneal administration for sustained photoacoustic contrast agent imaging

**DOI:** 10.1016/j.pacs.2022.100406

**Published:** 2022-09-26

**Authors:** Hailey I. Kilian, Chenshuo Ma, Huijuan Zhang, Maomao Chen, Anoop Nilam, Breandan Quinn, Yuqi Tang, Jun Xia, Junjie Yao, Jonathan F. Lovell

**Affiliations:** a Department of Biomedical Engineering, University at Buffalo, State University of New York, Buffalo, NY 14260, USA; b Department of Biomedical Engineering, Duke University, Durham, NC, USA

**Keywords:** Photoacoustic Imaging, NIR-II dye, Contrast imaging, Intraperitoneal, Intravenous

## Abstract

Photoacoustic (PA) imaging at 1064 nm in the second near-infrared (NIR-II) has attracted recent attention. We recently reported a surfactant-based formulation of a NIR-II dye (BIBDAH) for NIR-II PA contrast. Here, we investigated BIBDAH as a NIR-II PA contrast agent for longitudinal preclinical PA imaging. When administered to mice by the conventional intravenous (I.V.) route, BIBDAH was rapidly cleared from circulation, as indicated by a decrease in NIR-II absorption in sampled plasma. Conversely, when mice were injected with BIBDAH by the intraperitoneal (I.P.) route, peak NIR-II absorption levels in plasma were lower initially, but there was a sustained dye presence that resulted in a more consistent concentration of dye in plasma over 2 days. Increasing the I.P. injection dose and volume resulted in increased NIR-II area under the curve (AUC) in serum. Bimodal PA and ultrasound imaging reflected these results, showing a rapid decrease in PA signal in blood with I.V. administration, but permitting sustained imaging with I.P. administration. These results show that I.P. administration can be considered as an administration route in preclinical animal studies for improved longitudinal observation with more consistent contrast signal intensity.

## 1 Background

Photoacoustic imaging (PAI) is an emerging imaging modality with both preclinical and non-clinical usages. PAI is able to examine endogenous contrast in tissues including hemoglobin, lipids, melanin, and water [1], [2]. Moreover, one of the advantages of PAI is the capacity to image exogenous contrast agents at greater depths than other optical imaging modalities [3]. The second near-infrared window (NIR-II), from ∼1000–1700 nm, is appealing for PAI due to the lower attenuation and deeper penetration of light in tissues, leading to higher signal-to-noise ratio compared to shorter first near-infrared window (NIR-I) wavelengths [4], [5], [6], [7], [8]. While fluorescence imaging in the NIR-II window benefits from increased depth penetration, minimized tissue scattering, and diminished autofluorescence [9], PAI is also appealing since it relies on ultrasound (US) detection for image reconstruction, enabling imaging at much greater depths. A wide range of contrast agents have been employed for PAI [10], [11], [12], [13], [14], [15], [16], [17].

Administration of exogenous contrast agents for preclinical imaging is commonplace in PAI. Generally, to introduce agents directly into systemic circulation, the intravenous (I.V.) route is most commonly used. Routes of administration can include intraperitoneal (I.P.), subcutaneous, intranasal, intraocular, intramuscular, oral, sublingual, intrarectal among others. I.P. and I.V. administration of contrast agents have been compared [18], [19]. For I.P. administration, which is frequently used in preclinical research [20], drainage of the injected material into blood circulation is a delayed process that could be advantageous for PAI to enable a prolonged contrast imaging window, potentially with more consistent contrast concentrations in blood over time.

Vascular contrast imaging would benefit from a non-invasive approach, with a steady signal and extended imaging time window [21], [22]. This is particularly true for PAI, where the linearity and dynamic range of responses of imaging systems could be limited. Applications for vascular imaging include hepatic fibrosis diagnostics, brain imaging for psychiatric diagnostics, and cardiology [23], [24], [25]. In the current study, we demonstrate that in mice, by simply varying the administration route of an exogenous NIR-II contrast agent from I.V. to I.P., more consistent blood contrast is achieved with extended contrast duration.

## 2 Results

**Fig. 1:**
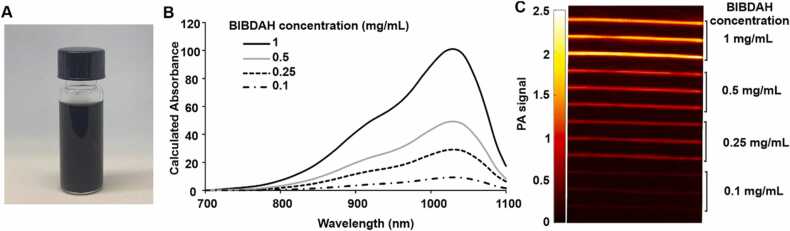
Facile preparation of BIBDAH as a NIR-II PAI contrast agent. (A) Photograph of a BIBDAH solution formulated in Kolliphor HS-15 surfactant in a glass vial. (B) Calculated absorption spectra of BIBDAH at varying concentrations. Calculated absorption is obtained from a dilute BIBDAH solution and is multiplied by the dilution factor. (C) PAI of an Intralipid phantom with tubes containing the indicated BIBDAH concentrations in PBS in triplicate. The phantom was placed at 4 cm depth away from the transducer.

The cyanine dye benzo indole butyl diphenylaminocyclopentene heptamethine (BIBDAH) was obtained commercially and prepared as previously described [26] by dissolving the dye in an aqueous solution of 25 % (w/v) HS15 Kolliphor surfactant in phosphate buffered saline (PBS). This resulted in the formation of darkly colored micelles (Fig. 1A). The BIBDAH formulation has a peak absorbance at 1036 nm with substantial contrast at the 1064 nm wavelength used by the Nd: YAG laser for PAI. The absorbance spectra of varying dilutions of BIBDAH are shown in Fig. 1B, demonstrating high NIR-II absorption. BIBDAH was shown to be active for PAI contrast imaging, as shown in Fig. 1C in a phantom that was imaged in water mixed with 2% Intralipid to mimic *in vivo* tissue scattering conditions [27]. Tubes were placed at 4 cm depth, to match the focal length of the transducer and as PAI phantoms containing NIR-II contrast agents can routinely image at multi-cm depths [28], [29], [30], [31].

**Fig. 2:**
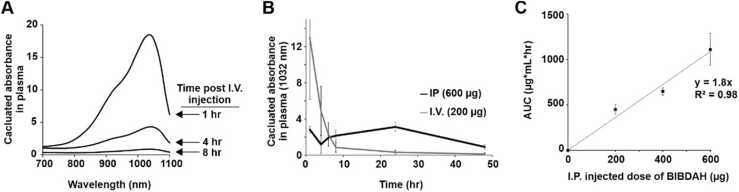
Pharmacokinetic profile of BIBDAH with I.V. or I.P. administration. (A) Absorbance of collected plasma following I.V. injection of 0.2 mg BIBDAH at the indicated time points following injection. (B) Absorption in collected plasma following for I.P. (0.6 mg) or I.V. (0.2 mg) injection of BIBDAH in mice. The higher permissible injection volume enabled more contrast agent to be administered with I.P. administration. (C) Area under the curve for varying doses of BIBDAH calculated for the period up to 48 h following I.P. injection.

Mice were then administered the BIBDAH formulation. For the preclinical usage of BIBDAH as a PA contrast agent, signal intensity, which depends on contrast agent concentration, is an important metric when imaging over a prolonged period of time, and impacts the ease with which longitudinal measurements to be carried out. In particular, blood contrast imaging was the focus of the study. Contrast measurements were taken by directly measuring the absorption of sampled plasma using a spectrophotometer. Fig. 2A shows there was a significant decrease in the calculated NIR-II absorption in plasma from the initial time point (1 h) to 8 h following bolus I.V. injection of 0.2 mg BIBDAH. This rapidly declining NIR-II absorption value, which decreased more than 15 fold in the period, reflects the physical clearance of the dye from blood into other organs. Such a significant decrease in contrast signal intensity could complicate PA contrast imaging at multiple time points within this period.

Given that I.P. administration appeared to provide a relatively consistent NIR-II contrast level in blood over time, the dose response was next assessed. This was done by I.P. injecting increasing doses of BIDAH and assessing the plasma area-under-the-curve (AUC) on the BIBDAH concentration vs. time plot over a 48-hour time period. The plasma kinetics of NIR-II contrast of both I.P. and I.V. injections are shown in Fig. 2B. The plots confirm that I.V. injection results in a rapid decline of blood contrast, whereas I.P. injection provides a sustained contrast level over 48 h. This gradual increase in plasma dye concentration can be accounted for by the delay it takes for BIBDAH to drain from the I.P. cavity into systemic circulation. The initial concentration of NIR-II contrast in blood was higher for I.V. administration. As shown in Fig. 2C, a roughly linear relationship was apparent between increasing I.P. injection doses of BIBDAH (from 0.2 to 0.4 to 0.6 mg)and the AUC in the blood. This is a desirable feature as it suggests a predictable behavior between the injection dose and resulting contrast signal.

Select pharmacokinetic parameters were further analyzed as shown in Table 1. The AUC parameter was comparable between 0.2 mg I.P. and I.V. injections, and was highest for the 0.6 mg BIBDAH dose. Higher I.V. dosages were not assessed since a 200 µL injection was not exceeded for I.V. administration due to limitations in permissible injection volume. The I.V. route provided the highest concentration of BIBDAH at the one hour time point (C_1hr_), being several fold greater than the I.P. route. However, by 24 hr, the level of BIBDAH in plasma for the I.V. route was lower than the I.V. route. The I.V. route plasma concentration of BIBDAH declined nearly 20 fold, whereas for the all I.P. doses provided for several more stable contrast levels in that period, with the 0.6 mg dose proving nearly uniform contrast between the 1 and 24 hr time points.

**Table 1:** Pharmacokinetic parameters.

Injection dose	AUC µg*h/mL	C_1hr_ µg/mL	C_24hr_ µg/mL	C_1hr_/C_24hr_ ratio
0.2 mg IV	638 (268)	130 (63)	7 (2)	19 (6.0)
0.2 mg IP	450 (55)	24 (5)	6 (2)	5 (2.2)
0.4 mg IP	649 (40)	29 (10)	16 (4)	2 (1.2)
0.6 mg IP	1110 (175)	31 (4)	33 (5)	0.9 (0.2)

Values in parentheses represent standard deviation from n = 4 biological replicates.

**Fig. 3:**
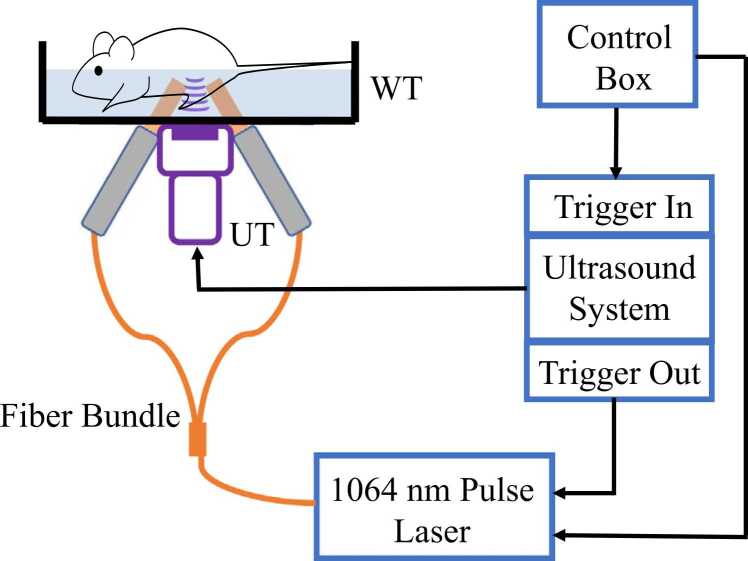
Schematic of the PAI system. UT: US transducer; WT: water tank.

Next, PA contrast imaging was carried out to further assess the behavior of I.V. and I.P. administration. The schematic of the PAI system is shown in Fig. 3. The excitation light was provided by an Nd:YAG pulse laser at 1064 nm (Qsmart; Quantel, Inc.) that was coupled into a fiber bundle and delivered to the imaging target through two linear fiber branches. The resultant ultrasound signals were detected by a 128-element linear array transducer L7–4 and acquired by a programmable ultrasound imaging system (Vantage256; Verasonics, Inc.). The excitation light pattern was aligned with the transducer focal plane. A bandpass filter ranging from 3 to 7 MHz was applied to the radio-frequency (RF) signals, and the tomographic PA images were obtained using the delay-and-sum reconstruction method. Meanwhile, US images were acquired with a single-element wobbler transducer mounted along side the linear array transducer [32], [33]. The axial focal length of the wobbler transducer was 19 mm, and the central frequency was 24 MHz. Rotational scanning of the wobbler was achieved by an encoded motor. PA and US images were co-registerted based on the relative positions of the linear array transducer and the wobbler. During PAI, the mice were anesthetized with 1.5 % v/v isoflurane and fixed on the imaging platform. The body temperature of the mice was maintained at ∼37 °C using a heating lamp.

**Fig. 4:**
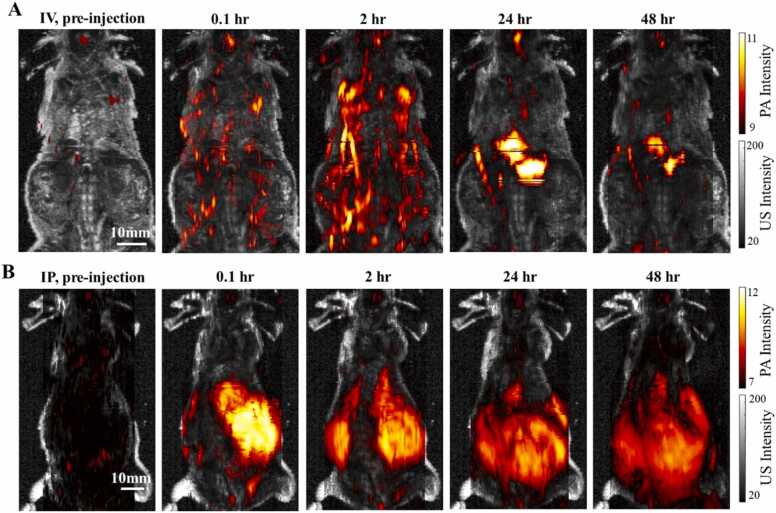
Co-registered whole-body PA and US MAP images of mice with I.V. and I.P. injection of BIBDAH. (A) Before I.V. injection, and post injection at 0.1 hr, 2 hrs, 24 hrs, and 48 hrs. (B) Before I.P. injection, and post injection at 0.1 hr, 2 hrs, 24 hrs, 48 hrs.

Fig. 4 shows the co-registered whole-body PA and US maximum amplitude projection (MAP) images of the mouse. The PA images were continuously acquired to monitor the BIBDAH distribution, while the US images provide anatomical structures of the mouse from the head to the tail. The PA and the US images shared the same imaging field of view (FOV). The PA images are presented in log scale to better show the signal distribution, while the US images are shown in linear scale.

The strong PA signal of BIBDAH was readily detected with both I.V. injection (Fig. 4A) and I.P. injection (Fig. 4B). The baseline images were acquired before injecting the dye. Only weak PA signals were observed, due to the relatively low optical absorption of the hemoglobin and other endogenous chromophores in tissues at 1064 nm. After injecting BIBDAH with a dosage of 0.2 mg via IV, PA images were acquired at 0.1, 2, 24 and 48 h (Fig. 4A). At 0.1 h, due to the strong NIR-II absorption of BIBDAH, the PA signals increased substaintially compared with the baseline image. The dye was then spread out to the whole body within the circulating system in the next few hours. After 2 h, the dye was distributed homogeneously in the major vessels and organs. By 24 h, PA signals from BIBDAH had largely diminished from the blood vessels while strong PA signals could be observed only at the liver region, suggesting the dye had accumulated in the liver. By 48 h, the dye signals had decreased in the liver, suggesting BIBDAH was further metabolized and cleared from the mouse body.

Fig. 4B shows PA images acquired before and at four different time points after I.P. injection of BIBDAH with a dose of 0.6 mg. Strong PA signal increase was detected locally in the abdominal cavity immediately after dye injection, indicating the pooling of the dye in the I.P. cavity. The dye signal decreased gradually over the next 48 h, as the dye was slowly transferred to the vasculature system. Therefore, PA imaging provides a mechanistic explanation for the sustained and moderated contrast in plasma observed following I.P. administration. Two hours after I.P. injection, PA signal was detected in a larger area, indicating the dye diffusion within the abdominal cavity. Over the next 48 hr, the PA signal in the abdominal cavity decreased continuously.

**Fig. 5:**
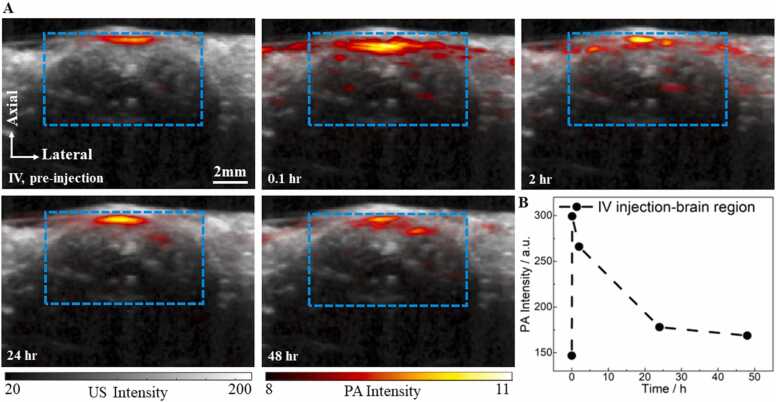
PAI of BIBDAH in the mouse brain vasculature with I.V. administration. (A) Co-registreted PA and US brain images before I.V. injection and post injection at different time points. (B) Averaged PA signal intensity in the brain at different time points. The dashed blue box indicates the region of interest for PA signal analysis.

Due to the interference of the strong BIBDAH signal around the I.P. injection area, it was challenging to study the circulation dynamics of BIBDAH in the vasculature system near the abdominal cavity. Therefore, BIBDAH imaging was also assessed in the mouse brain vasculature, which is far from the abdominal cavity and was not impacted by the interference from the initial injection. Imaging brain vasculature by photoacoustic tomography with the skull and skin intact is challenging, due to the optical attenuation and acoustic aberration by the skin and skull. Enhanced by the strong optical absorption in the NIR-II region of BIBDAH, PAT can enable brain vessels imaging even at relatively large depth, due to the improved contrast signal to noise ratio. Therefore, these experiments have demonstrated the potential in photoacoustic brain imaging with I.P. injection. Fig. 5A shows the co-registerted PA and US cross-sectional brain images. For PA signal analysis, we first applied a threshold of three times the noise level, estimated as the standard deviation of the background signals outside the target region. Then PA signals above the threshold were averaged to quantify the signal changes with I.P. and I.V. injections. PA signal analysis was performed over the same region of interest, as marked by the dashed blue box. In the baseline image before dye injection, weak background PA signals from hemoglobin were present in the cortex. After injecting BIBDAH with a dose of 0.2 mg via I.V., PA images were collected at 0.1, 2, 24 and 48 h. The dynamics of BIBDAH distribution in the brain vasculature were quantified by calculating the averaged PA signals above the noise level. At 0.1 h, the PA signal intensity increased by two folds in both the cortex and the deep brain. As BIBDAH was quicky cleared from the circulation over the following 48 h, the PA signals eventually decreased to 13% above the baseline (Fig. 5B).

**Fig. 6:**
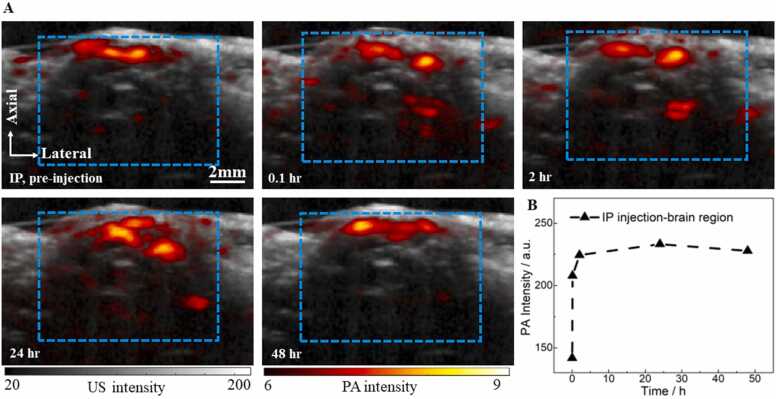
PAI of BIBDAH in the mouse brain vasculature with I.P. administration. (A) Co-registreted PA and US brain images before I.P. injection and post injection at different time points. (B) Averaged PA signal intensity in the brain at different time points. The dashed blue box indicates the region of interst for PA signal analysis.

Next, the distribution of BIBDAH in the mouse brain vasculature was assessed with I.P. injection. The co-registered PA and US images of the mouse brain vasculature are shown in Fig. 6A and the quantitative result was shown in Fig. 6B. About 2 h after I.P. injection with 0.6 mg of BIBDAH, the averaged PA signal intensity in the brain increased by 47%, compared with the baseline. Particularly, the PA signals in the deep brain were substaintially enchanced. Different from the quick signal drop with the I.V. injection shown in Fig. 5, the PA signals with the I.P. injection reached its maximum intensity after 24 h, and decreased only slightly after 48 h. The PA signal dynamics are consistent with the plasma pharmacokinetic measurements of BIBDAH, reflecting that BIBDAH concentration in the circulation system could be maintained consistently for at least a period of 48 h.

**Fig. 7:**
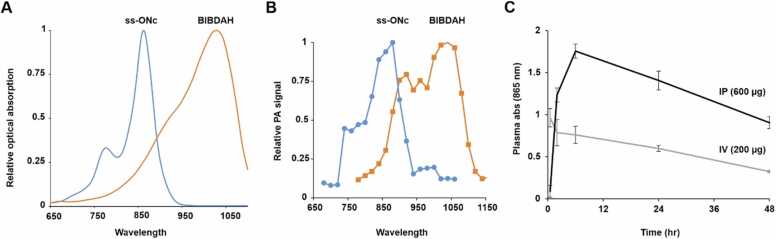
Characteristics of a second dye, ss-ONc for PA imaging. (A) Optical spectra and (B) PA spectra of ss-ONc and BIDAH in dilute PBS. (C) Plasma NIR optical absorption following I.V. or I.P. administration of ss-ONC to mice (n = 3). A higher permissible injection volume enabled more contrast agent to be administered with I.P. administration.

Next, to assess whether I.P. contrast administration could be beneficial for other dyes, we used surfactant-stripped Octabotoxynapthalocyanine (ss-ONc), a high contrast PA dye with peak absorption around 865 nm [34]. ss-ONC has blue-shifted peak optical absorption (Fig. 7A) and photoacoustic contrast (Fig. 7B) relative to BIBDAH. Mice were administered ONc and plasma was sampled. As shown in Fig. 7C, I.P. administration gave rise to a delay in the peak plasma absorption until the 6 h time point, compared to I.V. administration which decreased immediately, similarly to behavior of BIBDAH. I.P. administration permitted a higher injection volume of contrast which led to a greater AUC and also higher NIR absorption contrast in plasma throughout the imaging period.

## 3 Discussion

PAI is capable of imaging major blood vessels of deep internal organs *in vivo*. Although the NIR-II light has a larger penetration depth in tissue than visible light, the detection sensitivity of PAI can be limited due to weak optical absorption of blood in this wavelength range. By taking advantage of the strong optical absorption at 1064 nm, BIBDAH can improve the penetration depth of PA images. Based on plasma sampling, we found that administation of BIBDAH by I.P. route resulted in a longer circulating period and more consistent dye concentration in blood relative to the I.V. administration. The dose of BIBDAH with I.P. administration was roughly proportional to the NIR-II AUC in blood. Whole-body PA and US images of the mice with I.V. and I.P. injections not only confirmed the pharmacokinetic findings, but also provided more details of the dye behavior, such as the accumulation of BIBDAH in the liver following I.V. administration and the pooling in the abdominal cavity following I.P. administration. The co-registered PA and US images proved useful for gauging accumulation locations and dynamic changes of the dye at different time points.

For the I.V. injected mouse, the dye was quickly distributed to the whole body through the blood circulation of the vasculature system after the injection. We observed significant PA signal enhancement right after the injection, as shown in Fig. 4A. The distribution of the dye can be observed from the PA images acquired at three different post-injection time points. The dye firstly spread out to the whole body in 2 h, accumulated in the liver in 24 h, and was then likely excreted from the body by 48 h or was further degraded. The behavior of the dye was also studied in the mouse brain. The PA images of the brain vasculature (Fig. 5A) and the quantitative dynamics (Fig. 5B) showed similar trends, comparing with the signal changes obtained from the plasma samples. The PA signal enhancement depends on the region of interest and the time. Generally speaking, the SNR with IV injection is better than IP in a short time period post injection, but decays faster. The signal enhancement by IP injection can last for at least two days while less than one day for IV injection. These results demonstrated that the dye got into the vasculature quickly after I.V. injection at a high concentration, but was then rapidly removed from circulation. Compared to I.V. injection, BIBDAH showed different dynamics with I.P. injection. The PA signals in the abdominal cavity remained strong even after 2 days (Fig. 4B). This same dynamics were also observed with brain imaging depicted in Fig. 6A and the quantitative results shown in Fig. 6B. The PA signals increased immediately after I.P. injection, reached maximum after 24 h, and stayed at high levels even after 48 h, showing that the dye was transferred gradually from the abdominal cavity to the blood vessels and continuously circulated to the whole body with the blood flow.

This study points to several advantages of the I.P. route of contrast administration. The extended blood circulation, due to the time needed for drainage into circulation, would be advantageous where longitudinal imaging is used. A narrower range of contrast level also potentially enables instrumentation to be used without changing gain or other acquisition settings. Another advantage of the I.P. route relative to I.V. is that in preclinical studies, a greater permissible injection volume enables more contrast agent to be delivered, which in most cases is desirable. We did not observe any toxic effects of the dyes during the multiple-day imaging period, however a toxicity study could be carried out to determine if the higher contrast dosage enabled by I.P. administration would lead to great toxicity, based on the effects of the contrast agent itself. While we previously reported that BIBDAH is primarily taken up by the liver as it leaves circulation [26] following I.V. administration, assessing how this might be modulated by I.P. administration would be useful. Finally, in mice, I.P. injections are easier to carry out compared to I.V. injections, which could be another reason that I.P. injections should be considered for PA contrast administration in preclinical imaging studies.

The results presented here were limited to two PAI contrast agents that were formulated with a biocompatible surfactants. It is not clear whether I.P. administration would have similar effects with other types and formulations of PA contrast agents. Likewise, we did not explore whether other animal species would also benefit from I.P. administration for sustained contrast imaging. While the I.P. route of administation may be useful to consider for small animal contrast PAI, it is not clear whether this approach has any relevance to eventual application of PA contrast agent administration for humans. In mice, this study demonstrated the feasibility of brain vasculature imaging with I.P. contrast administation, research which could be extended in the future to assess PAI imaging in various preclinical neurological disease models such as stroke, traumatic brain injury, or glioblastoma. All there avenues of research would be interesting areas to examine in the future.

## 4 Conclusion

The route of administration is an important parameter to consider for pharmaceutical formulations. Likewise, contrast agents as well will exhibit different biodistribution and pharmacokinetic behavior based on the administration route. In the present study, we show that in mice, I.P. administration of a simple-to-produce NIR-II PA contrast agent leads to advantageous features compared to I.V. administration for PA vasculature imaging. Notably, there was less range in NIR-II contrast intensity over time after I.P. administration, while I.V. bolus administration resulted in a high initial maximum blood concentration that quickly decreased. Furthermore, NIR-II contrast levels were sustained for a longer duration of at least multiple days. Co-registered PA and ultrasound imaging was useful for observing contrast distribution *in vivo* by providing molecular and anatomical information.

## 5 Experimental

### 5.1 Materials

BIBDAH-BF4 was purchased from Spectrum Info LTD (Catalog # S01451). Kolliphor HS15 (#42966) and Intralipid® were purchased from Sigma-Aldrich. BIBDAH was prepared by dissolving it in 137 mM NaCl pH 7.4 PBS containing 25 % w/v Kolliphor HS15 with sonication at 60 °C for 30 min. The final concentration of BIBDAH was 1 mg/mL. The BIBDAH solution was passed through a 0.2 µm sterile filter membrane from Thomas Scientific, Catalog #1190M42 prior to usage. BIBDAH micelle size was 12 nm with a polydispersity index of less than 0.1, based on dynamic light scattering as measured in dilute PBS in a Nanobrook 90 plus PALS instrument. Surfactant-stripped ONc was produced as previously described [34].

### 5.2 Dye absorption and pharmacokinetic characterization

For dye characterization at varying dilution, 10 µL of the BIBDAH formulation was diluted into 990 µL of PBS for measurement in a spectrometer (Lambda 365 UV/Vis by PerkinElmer). The dilution factor was factored back in when plotting the calculated absorption. For animal studies, all protocols were approved by Institutional Animal Care and Use Committee (IACUC) of University at Buffalo. 6–8 week old female outbred ICR mice (Envigo) were used for the experiments. I.V. injection was by lateral tail vein and I.P. injection was through the abdominal surface into peritoneal cavity with 27G and 29G needles for I.P. and I.V. respectively. Following administration, blood from the saphenous vein was collected in EDTA tubes (Thermo Fisher Scientific # NC9141704). at the specified time points, centrifuged at 3000 rpm for 15 mins, then the plasma was collected. Each whole blood collection volume was ∼30 µL, and plasma final volumes typically ranged from 5 to 20 µL. Plasma was diluted into a 200 µL cuvette and the dilution factor was recorded and used for calculating the total NIR-II absorption. If 5, 10, or 15 µL of plasma was collected then 195, 190, or 185 µL of PBS was used respectively, to provide a dilution factor of 40, 20, or 13.3× for calculations. The wavelength used for analysis was 1032 nm and concentrations were determined with a standard curve. To assess pharmacokinetic parameters, for each pharmacokinetic data set, area under the curve (AUC) and concentration parameters (e.g. C_max_) were determined from the data plots using trapezoid calculations for AUC. ss-ONc was assessed in a similar manner, using wavelength measurements at 860 nm.

### 5.3 PA imaging in vitro

The photoacoustic excitation source was a 10-Hz pulsed Nd:YAG laser (Continuum Surelite III) with output wavelength of 1064 nm. A 128-element linear transducer array (Imasonics, Inc) with central frequency of 2.25 MHz was used as the PAI detector. The PAI data was acquired using the Vantage Verasonics data acquisition system. For tube phantom experiment, a 3D model was made in Solidworks to place the plastic tubes. In this model, holes were made for 2 mm tubing with 1 mm inner diameter, spaced 5 mm apart and printed by a 3D printer (Uprint by Stratasys), similar to a previously reported approach.[26] BIBDAH-BF4 in 25 % HS25 at the concentrations of 0.1, 0.25, 0.5, 1.0 mg/mL were injected into tubes in sequence for PAI. The tubes were closed off by knotting the ends. The prepared tube phantom was then immersed in a water tank mixed with 2 % Intralipid to mimic optical tissue scattering and scanned linearly at the speed of 1 mm/s controlled by the translation stage. The laser output energy was measured as 25 mJ/cm^2^. The acquired raw data was reconstructed using a back-projection algorithm in MATLAB.

### 5.4 Co-registered PA and US imaging *in vivo*

For *in vivo* PAI, a pulsed 1064 nm laser (Q-smart 850, Quantel) was used as the excitation light source, and the pulse repetition rate was 10 Hz. The light was delivered to the animal through a fiber bundle. The generated PA signals were detected by a 128-element linear array US transducer (L7–4, Philips) and collected by a programmable US imaging system (Vantage 256, Varasonics). The cross-sectional PA images were acquired at the frame rate of 10 Hz. By scanning across the mouse along the elevational direction using a motorized translation stage, 3D volumetric images were obtained. The collected PA signals were first filtered using a bandpass filter and then reconstructed using the delay-and-sum method.

For US imaging, a single-element focused wobbler ultrasound transducer (Vega, SonoVol) with a central frequency of 24 MHz was used. The wobbler was mounted on the same translation stage as the linear array transducer for PAI. The focal length of the transducer was 19 mm, and the lateral scanning range was 2.1 cm. The transducer’s scanning spatial step size was 0.25 mm. A 3D volumetric US image was acquired by fast-scanning of the wobbler and slow-scanning of the motorized translation stage. The 3D PA and US images can be readily co-registered based on the physical distance of the linear array transducer and the wobbler.

*In vivo* PA and US imaging were performed with the approval of the Institutional Animal Care and Use Committee (IACUC) of Duke University. Swiss webster mice at the age of 12-weeks with the body weight of ∼25 g were used for the experiments. The mice were anesthetized with 1.5 % isoflurane and fixed on the imaging platform. The body temperature of the mice were kept at ∼37 °C using a heating lamp during the whole imaging process.

## Declaration of Competing Interest

The authors declare that they have no known competing financial interests or personal relationships that could have appeared to influence the work reported in this paper.

## Data Availability

Data will be made available on request.
